# 3D printing of radioactive wall-less PET phantoms improves threshold-based target delineation and quantification

**DOI:** 10.1186/s40658-025-00768-x

**Published:** 2025-06-06

**Authors:** Adrian Jun Zounek, Nico Maximilian Joerg, Felix Lindheimer, Artem Zatcepin, Giovanna Palumbo, Rosel Oos, Astrid Delker, Franz Josef Gildehaus, Andreas Bollenbacher, Guido Boening, Peter Bartenstein, Matthias Brendel, Nathalie Lisa Albert, Sibylle Ziegler, Lena Kaiser

**Affiliations:** 1 Department of Nuclear Medicine, LMU University Hospital, LMU Munich, Marchioninistr. 15, 81377 Munich, Germany; 2 German Center for Neurodegenerative Diseases (DZNE), 81377 Munich, Germany; 3 Partner Site Munich, A Partnership Between German Cancer Research Center (DKFZ) and LMU Munich, German Cancer Consortium (DKTK), Munich, Germany; 4 Bavarian Cancer Research Center (BZKF), Partner Site Munich, Munich, Germany; 5 Munich Cluster for Systems Neurology (SyNergy), Munich, Germany; 6 Department of Nuclear Medicine, Hannover Medical School (MHH), Hannover, Germany

**Keywords:** Segmentation, Phantoms, 3D printing, PET

## Abstract

**Background:**

Validation of threshold-based PET segmentation and PET quantification is typically performed with fillable phantoms. Theoretical considerations show that the inactive walls of the phantom cavities introduce a contrast dependence of the volume-reproducing threshold (VRT), potentially leading to segmentation errors and therefore miscalculations of target volumes. The goal of this study was to experimentally show the contrast independence of the VRT when using wall-less phantoms.

**Results:**

Radioactive spheres were produced according to NEMA specifications (D = 10/13/17/22/28/37 mm) using a stereolithographic (SLA) 3D printer. For comparison, hollow spheres were filled with a similar activity concentration. Image data from both sphere types were acquired with five different signal-to-background ratios (SBR = 2/4/6/8/10) using a Siemens mCT 20 and a Biograph 64 TruePoint PET/CT system. Results from wall-less and fillable spheres were compared to evaluate contrast dependence and segmentation accuracy based on VRT and intensity profiles. Wall-less phantoms demonstrated consistent VRT values, with a coefficient of variation of 2% over all SBRs, indicating independence from contrast. Conversely, fillable phantoms exhibited significant VRT variability, with a coefficient of variation (CV) of 9% over all SBRs and up to 40% volume overestimation at low contrast. Additionally, activity distribution in the printed spheres was evaluated using PET-based statistical analysis and autoradiography. The PET intensity distribution in the printed material was highly uniform (CV = 4.2%), with a Kullback–Leibler divergence near zero and no statistically significant difference to the fillable spheres. Autoradiography revealed microscopic regions with elevated counts, showing a CV of 11.7%, which was effectively reduced to 2.4% after Gaussian filtering.

**Conclusions:**

The theoretical predictions of a significant influence of inactive walls in low-contrast images and contrast-independent VRT in wall-less phantoms were successfully confirmed. SLA 3D printing of phantoms is a promising method for the reliable evaluation of PET quantification methods, particularly in low-contrast scenarios commonly encountered in clinical settings.

**Supplementary Information:**

The online version contains supplementary material available at 10.1186/s40658-025-00768-x.

## Introduction

In oncology, pathological tissues such as tumors can be detected using PET. Accurate target volume delineation is critical for effective treatment planning, particularly in radiotherapy where the delivery of therapeutic doses depends on the precise definition of tumor margins. Furthermore, monitoring the extent of the disease is crucial for evaluating treatment response and for subsequent treatment decisions. Traditional methodologies for the validation of segmentation algorithms primarily rely on the use of fillable phantoms like the Hoffman 3D brain phantom [1] or the NEMA IEC PET body phantom [2, 3]. Various activity distributions can be generated by filling the phantom compartments with liquid compounds spiked with radiotracers to mimic the distribution within a patient. The design and production of these phantoms is usually carried out by specialized manufacturers according to international standards.

However, previous studies have highlighted a significant limitation associated with fillable phantoms. In particular, inactive walls separating the phantom compartments introduce a contrast dependence of the volume-reproducing threshold (VRT) [4–6]. This potentially diminishes the accuracy of threshold-based methods, which represent one of the most popular image segmentation methods in PET [7, 8], potentially leading to miscalculations of target volumes.

Several alternative techniques for phantom manufacturing based on materials such as wax, gelatin, paper, cryogel, alginate, and epoxy resin have been explored to overcome this limitation [9–16], among which stereolithographic (SLA) 3D printing technology stands out as a promising solution [17–21]. This technique features high flexibility and facilitates the fabrication of intricate massive objects with arbitrary shapes that more accurately replicate the clinical scenarios encountered in diagnostic nuclear medicine.

Our study builds upon the theoretical groundwork of Hofheinz et al. [4] and the practical applications by Berthon et al., Meier et al., Läppchen et al. and Gillet et al. [6, 17–19]. We aim to empirically assess whether the use of 3D printed wall-less phantoms facilitates improved PET segmentation validation compared to conventional fillable phantoms by eliminating drawbacks such as the contrast dependence of the segmentation threshold.

## Methods

### Phantom design and production

3D models of spheres were designed in the CAD software Fusion 360 (ver. 2.0.18220, Autodesk, Mill Valley, USA) according to NEMA body phantom specifications with diameters of 10, 13, 17, 22, 28, 37 mm [2]. Slices for SLA printing were generated from the models using PrusaSlicer (ver. 2.6.0, Prusa Research, Prague, Czech Republic).

After drawing up the desired activity of [^18^F]FDG in a syringe using a dose calibrator, the compound was injected into the liquid printing resin (Prusament Resin Model Transparent Amber, density: 1.09 g/cm^3^) and homogenized with a magnetic stirrer for 15 min. The resin contains cyclic trimethylolpropane formal acrylate, urethane diacrylate, tricyclodecane dimethanol diacrylate, and ethoxylated pentaerythritol tetraacrylate in an unspecified ratio according to the manufacturer [22]. The mixture was transferred to the Prusa SL1S 3D printer by pouring it into the resin tank. All spheres were printed simultaneously using a layer height of 0.1 mm, resulting in a print duration of approximately 70 min.

**Fig. 1:**
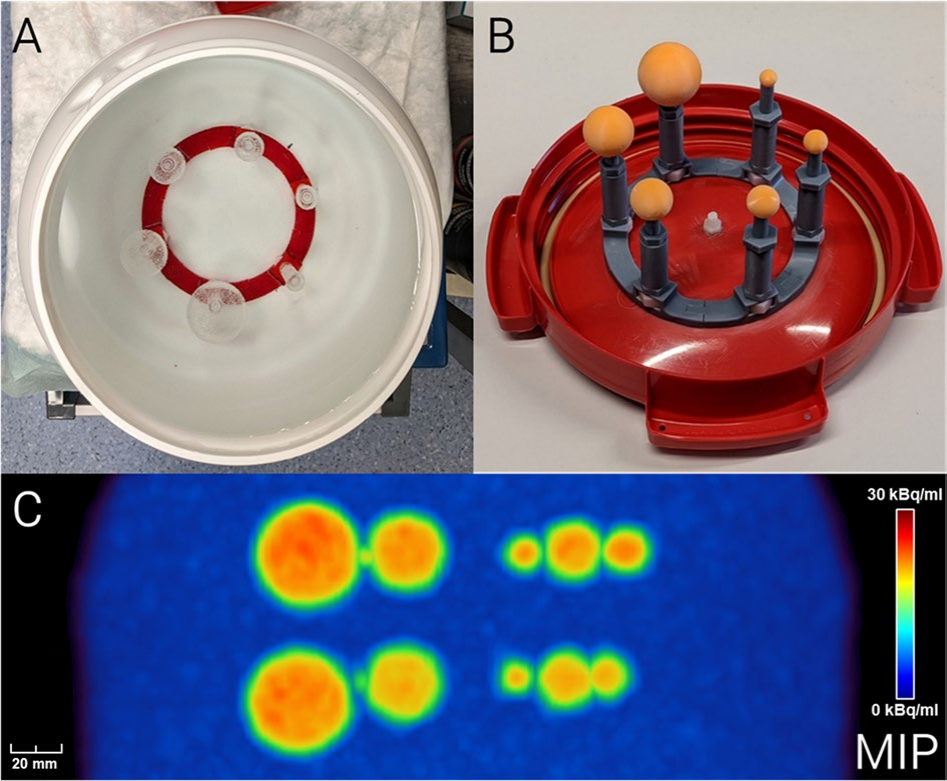
**A** Fillable NEMA phantom spheres mounted to bottom of phantom container. **B** 3D printed wall-less spheres mounted to lid of container. **C** Maximum intensity projection of high contrast PET image (signal-to-background ratio: approx. 9.5)

Next, the support structures were removed, and the spheres were sanded to obtain a smooth surface. The spheres were washed with isopropyl alcohol for 5 min and cured with UV light for 3 min using the dedicated Prusa CW1S washing and curing station and adhering to the manufacturer’s recommendations. In order to replicate the spatial arrangement of the spheres in the NEMA phantom, the 3D printed spheres were mounted on a custom ring-shaped 3D printed scaffold using a water-resistant liquid adhesive. The scaffold including the spheres was fixed to the lid of a watertight container to enable measurements with background activity (Fig. 1).

The NEMA spheres were removed from the original phantom, filled with a mixture of water and [^18^F]FDG, and fixed to the bottom of the container opposite to the 3D printed spheres. In this configuration, the printed spheres and the fillable spheres could be measured at the same time with identical background activity.

### Image acquisition

Before image acquisition, the phantom was filled with a mixture of water and [^18^F]FDG to obtain non-zero background activity. Low-dose CT for attenuation correction and PET listmode data were then acquired on a Biograph 64 TruePoint as well as a Biograph mCT 20 PET/CT system (Siemens Healthineers AG, Erlangen, Germany) at the Department of Nuclear Medicine of the University Hospital of LMU Munich. The phantom was scanned on the second device immediately after the first acquisition. The two scans were repeated four additional times after adding activity to the background to obtain five different signal-to-background ratios (SBR) for each type of sphere, with acquisition times ranging from 5 to 25 min.

An automatic gamma counter (Hidex GmbH, Mainz, Germany) was employed to measure the activity concentrations in the spheres and the background. The samples for the gamma counter measurements were obtained by pipetting the water and the resin that was used for the phantom and by 3D printing suitable objects along with the spheres. All PET data were reconstructed using 3D ordered subsets expectation maximization (OSEM3D) with a voxel size of 1 × 1 × 2 mm^3^, a 5 mm full width at half maximum (FWHM) Gaussian smoothing filter, and an effective number of 84 subsets (Biograph 64: 4 iterations, 21 subsets; Biograph mCT 20: 7 iterations, 12 subsets). Standard corrections for random and scattered coincidences, radionuclide decay, photon attenuation and detector dead time were applied during reconstruction. These parameters correspond to the reconstruction settings that are used in clinical routine for the evaluation of gliomas at our department. Furthermore, the reconstructed frame durations selected from the listmode data were adjusted to match the detected photon counts originating from the spheres between the different SBR acquisitions. Figure 1 shows photographs and a PET image of the assembled phantom.

### Segmentation and preprocessing

First, volumes of interest (VOI) were defined manually to determine the background signal and to locate the spheres using MITK Workbench (ver. 2021.10 [23]). The SBRs of the subsequent image acquisitions were determined by defining a VOI with a diameter of 20 mm in the center of the 37 mm sphere and dividing its mean activity concentration by the mean activity concentration in the background VOI. The following SBRs were achieved: 9.3/8.4/6.5/4.3/2.0 for the fillable spheres and 9.8/8.7/6.7/4.3/2.0 for the printed spheres. Seed voxels were assigned to each sphere to initialize the region growing segmentation algorithm from the SimpleITK library (ver. 2.2.1 [24]) in Python (ver. 3.10) using the included ConnectedThreshold function. The real volumes of the spheres were known due to the defined dimensions of the printed and the fillable spheres. Thus, the absolute volume-reproducing threshold T could be determined iteratively by minimizing the difference between the segmented volume and the real volume. The Hounsfield units (HU) of the printed material were determined by extracting the mean signal of the largest wall-less sphere from one of the CT acquisitions. Finally, the Hounsfield values were converted to the linear attenuation coefficient for 511 keV photons using the transformation described by Burger et al. [25].

### VRT calculation

The background-corrected relative volume-reproducing threshold VRT, which is independent of the sphere activity A and the background activity B, was calculated according to the formula VRT = (T-B)/(A-B) as described by Jentzen et al. [5] and Hofheinz et al. [4]. VRT values for the 3D printed and the fillable spheres, as well as the percentage difference, were plotted against the five different SBRs for the six sphere sizes to show the effect of inactive walls. The coefficient of variation (CV) over all SBRs was calculated for each sphere size to characterize the contrast dependence of the VRT. Furthermore, the VRT values from the fillable spheres were used to segment the wall-less spheres to quantify volume overestimation. This was achieved by calculating the corresponding absolute threshold T and using it as an input for the region growing algorithm on the images of the wall-less spheres.

### Intensity profiles

For the generation of intensity profiles, the spheres were segmented on the PET images by manually placing spherical VOIs with the known sphere dimensions and expanding them to include an additional margin of the surrounding area. Next, the center-of-mass voxel was determined using the Python package SciPy (ver. 1.10.1, [26]). Intensity profiles were generated by plotting all voxel intensities against the radial distance with respect to the center voxel. The plots were mirrored to create a symmetrical image.

### Analysis of radioactivity distribution

The PET signal distribution inside of the largest printed and fillable spheres was quantified for the highest SBR by generating spherical VOIs with a diameter of 27 mm around the respective center-of-mass voxels to rule out spill-in and spill-out effects and by calculating the CV. Additionally, the Kullback–Leibler divergence (KL) and the Kolmogorov–Smirnov test statistic (KS) were calculated to quantify the difference between the normalized voxel distributions.

A cuboid object with the dimensions 60 × 20 × 1 mm was printed after mixing the same resin that was used for the spheres with [^18^F]FDG as described above. The object was placed in a cassette with a phosphor image plate for overnight exposure. A computed radiography reader was used in conjunction with the AIDA Image Analysis software (ver. 4.50.010, Elysia-Raytest GmbH) to obtain a 25 µm high-resolution digital image from the plate, which was cropped to match the object outline. Finally, 5 mm FWHM Gaussian smoothing was applied to achieve the same filtering that was used in the reconstruction of the sphere phantom PET data. 3D surface plots were generated from the original image as well as the blurred image to visualize the 2D distribution of detected counts.

## Results

### 3D printing observations

The CAD designs of the phantom scaffold were reproduced with high precision using the 3D printer after producing a few prototypes. However, the first non-radioactive test prints of the spheres showed deformations along the vertical printing axis, leading to an ellipsoid shape of the sides that were closer to the print plate. This was successfully compensated in the subsequent prints by increasing the stability of the support structures in the slicing software. Thus, the removal of the stronger supports required longer and more complex handling (approx. 30 min) of the printed parts due to the increased thickness and support head diameter. Additionally, the hemisphere surfaces had to be sanded to smooth out the protruding remnants of the supports.

### Image segmentation

This section contains the results that were calculated from the Biograph mCT 20 PET/CT data. The data from the Biograph 64 TruePoint scanner is visualized in Fig. S1 of the supplementary material.

**Fig. 2:**
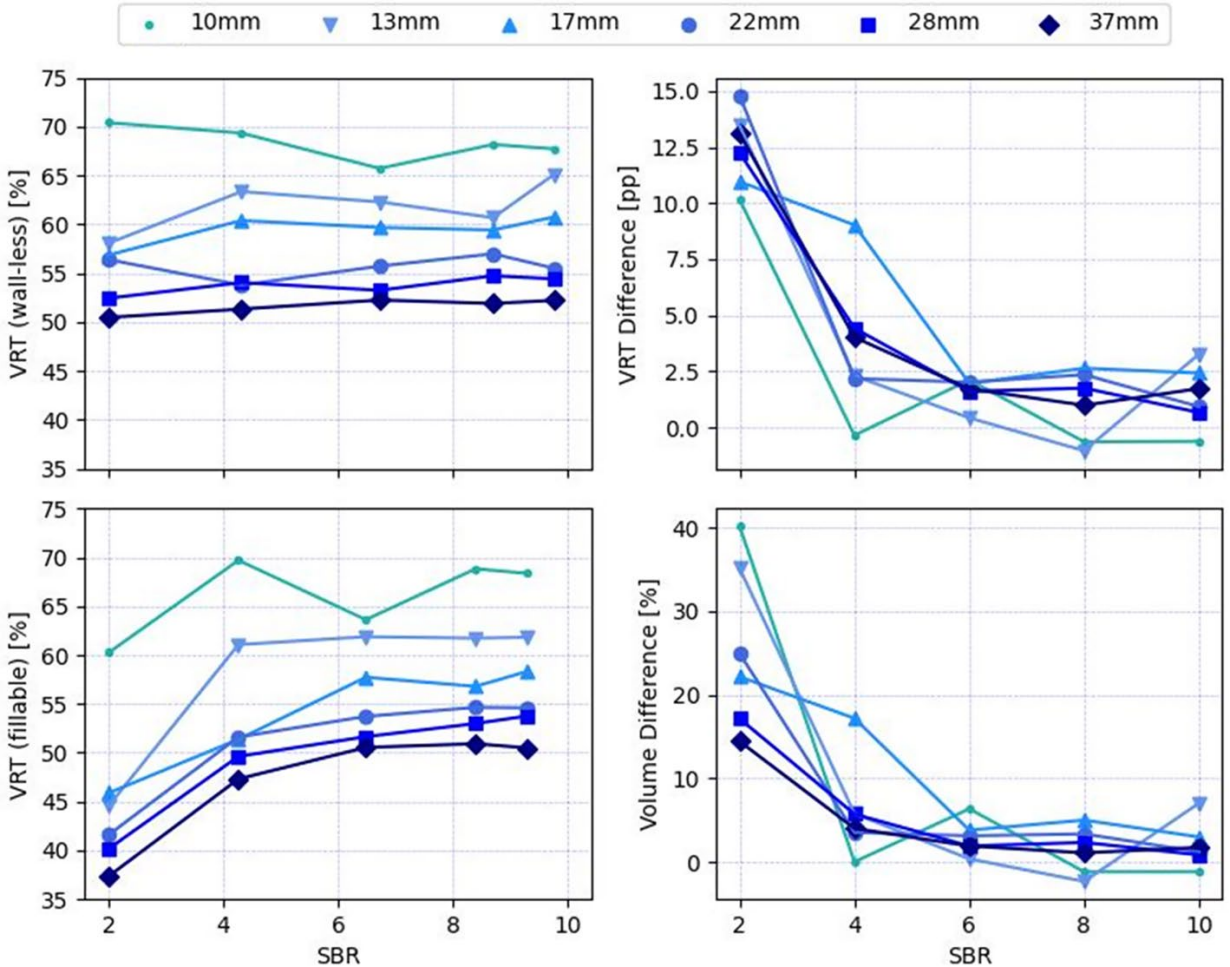
Line plots of volume-reproducing threshold (VRT) against signal-to-background ratio (SBR) for 6 sphere diameters, VRT difference between wall-less and fillable spheres, and the resulting difference of segmented volumes. pp: percentage points

**Fig. 3:**
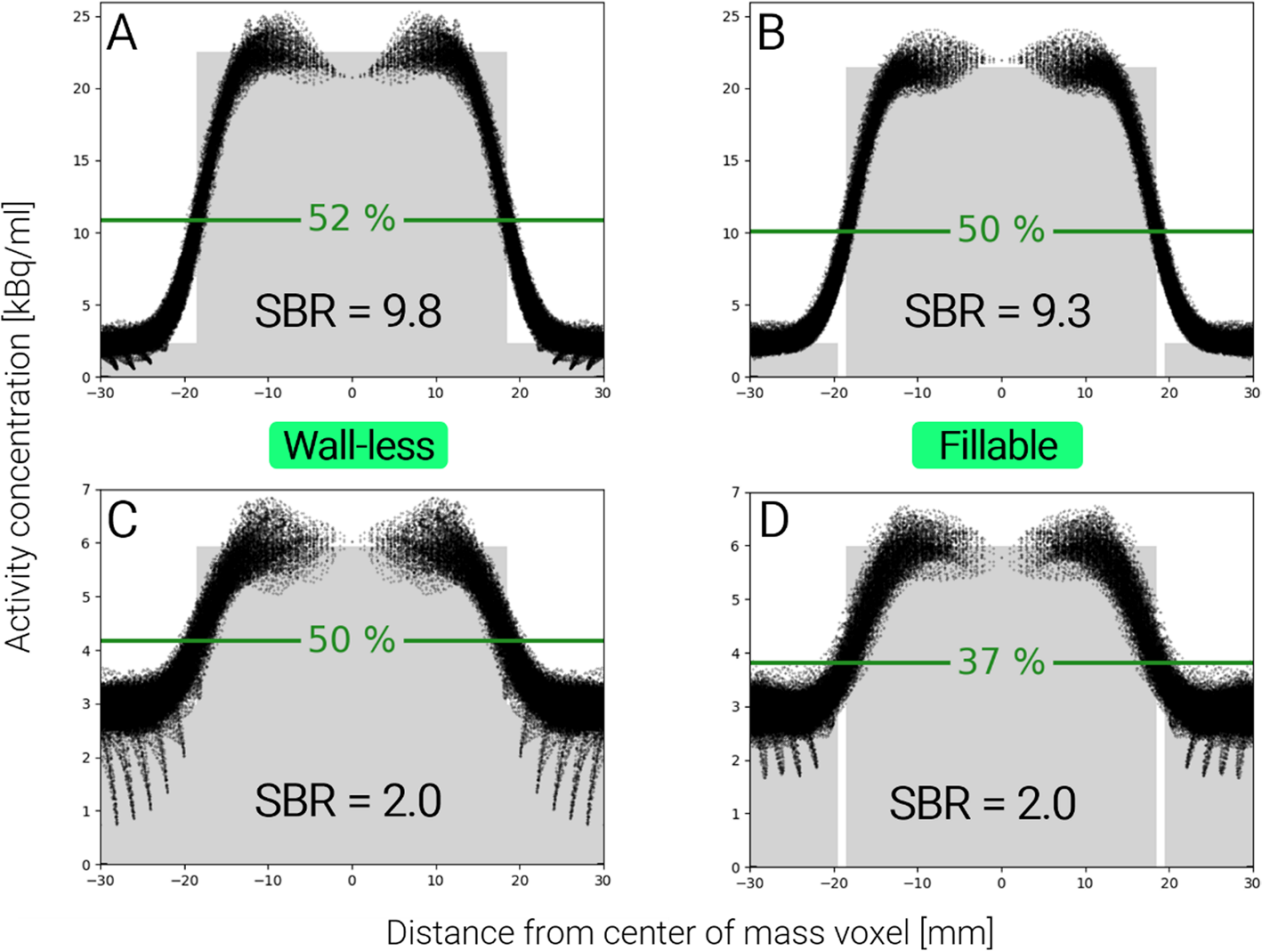
Intensity profiles of the largest spheres (⌀ 37 mm) at high (**A** & **B**) and low contrast (**C** & **D**) with a horizontal line marking the respective VRT. The grey area corresponds to the true measured activity concentration

The CT data yielded a mean value of 153 HU for the largest wall-less sphere, which corresponds to a linear attenuation coefficient of 0.105 cm^−1^ for 511 keV photons. Figure 2 depicts the VRT plotted against SBR. For the wall-less spheres, the VRT values ranged from 50 to 70% with a mean CV over all SBRs of 2%, resulting in relatively straight and horizontal line plots. Here, the CV was first calculated over all SBRs for each individual sphere, and then averaged over all sphere sizes. For the fillable spheres, the lines are curved downwards, i.e., a decreasing VRT was observed for low contrast, with values ranging from 37 to 70% with a mean CV of 9%. The largest VRT differences of up to 15 percentage points (pp) between the wall-less and the fillable spheres were observed for the lowest SBR, while the smallest differences with up to 3 pp were observed for the highest SBR. These two cases are visualized in the intensity profiles in Fig. 3 for the largest and smallest spheres.

**Fig. 4:**
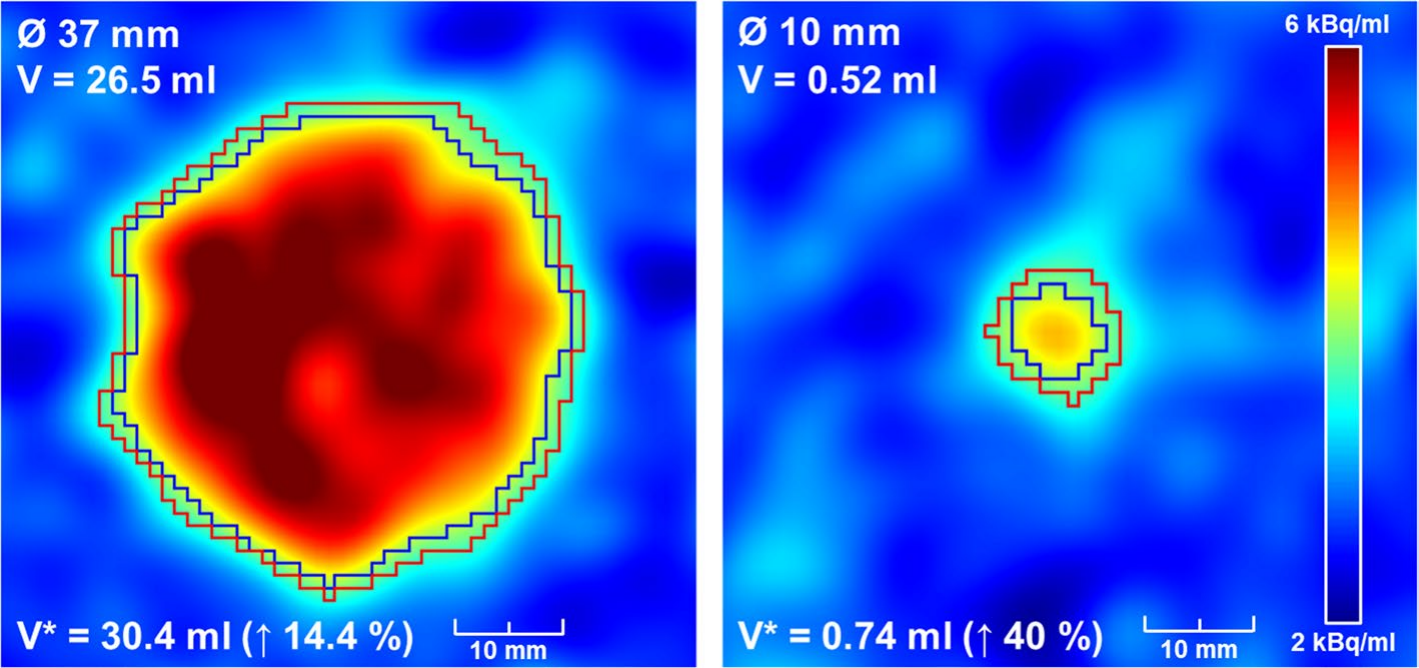
Axial PET slices showing the largest and smallest wall-less spheres as imaged at low contrast (signal-to-background ratio of approx. 2). The blue contour depicts the segment that reproduces the real sphere volume V. The red contour with volume V* was determined using the fillable spheres and leads to an overestimation of the real volume

Figutre 2 also shows the overestimation of the sphere volumes resulting from the application of the VRT that was derived from the fillable spheres on the images of the wall-less spheres. The average volume overestimation was 2% for the highest contrast. Figure 4 shows examples of image slices from the low contrast case, where the average overestimation was 26%, and the volume of the smallest sphere was overestimated by 40%. This is also reflected by the strongly curved line plots.

### Radioactivity distribution

**Fig. 5:**
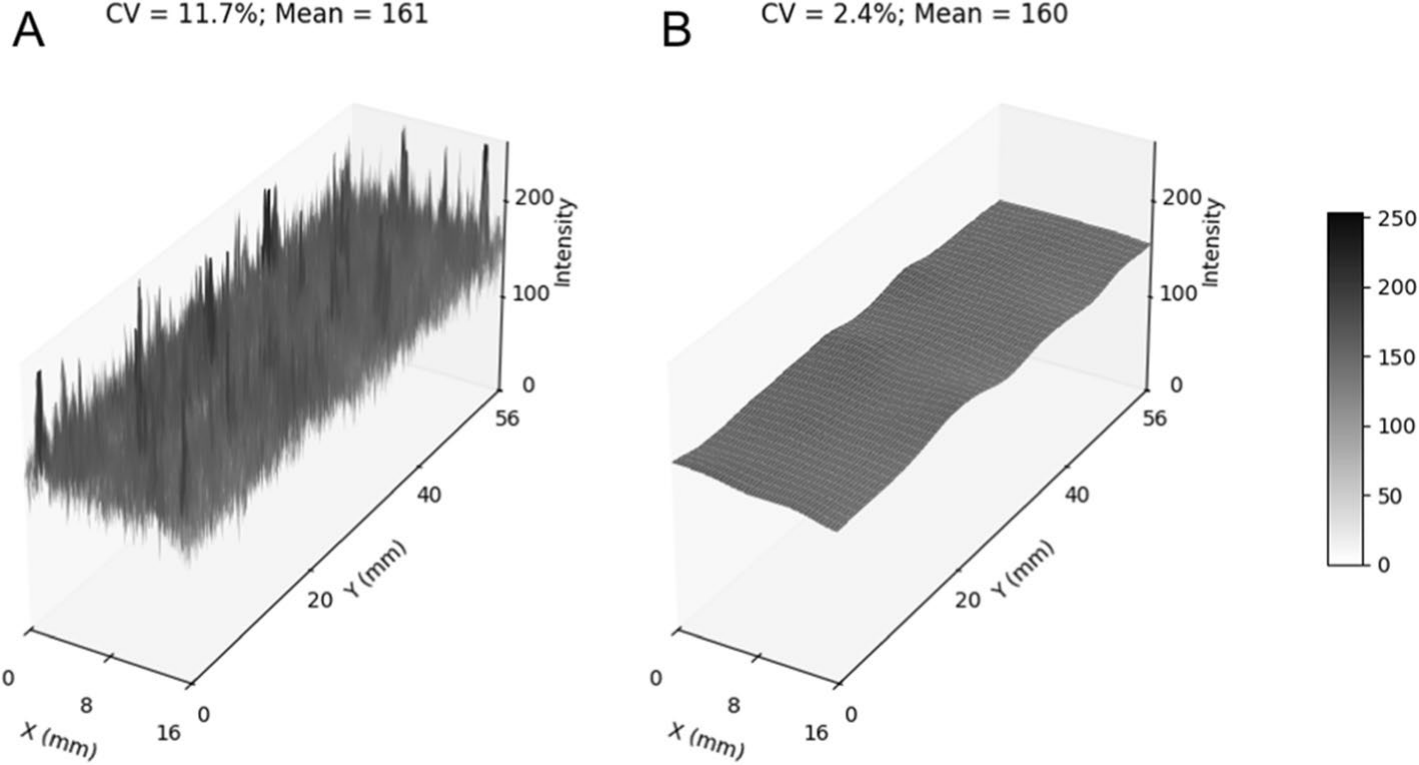
3D surface plots obtained from the autoradiography measurement of a 3D printed cuboid with 1 mm thickness including mean intensity and coefficient of variation (CV). **A** Raw high-resolution (25 µm) image. **B** Smoothed image after application of a 5 mm FWHM Gaussian filter

The analysis of the PET signal distribution in the center of the largest spheres yielded the following metrics: CV_3D_ = 4.2%; CV_fillable_ = 4.1%; KL = 0.02; KS = 0.02 (*p* = 0.11). Figure 5 depicts the distribution of counts from the autoradiography measurement before and after applying Gaussian smoothing.

## Discussion

In this work, we applied the novel methodology of 3D printing radioactive objects to evaluate the impact of inactive walls on threshold-based segmentation. Our results show that the segmentation threshold VRT is virtually constant for wall-less spheres at different SBR values, which implies that it is independent of the image contrast. Furthermore, our data clearly illustrates the size dependence of the VRT for spherical objects. In our case, the VRT was calculated from the mean activity concentration and therefore decreased with sphere size (see Fig. 2). We were also able to observe these tendencies in the data from the second PET scanner, which emphasizes the robustness of the results. This is in good agreement with the findings of Hofheinz et al. [4] and Jentzen et al. [5], who previously published their theoretical considerations regarding wall-less objects. Although the SBRs of the different sphere types were not matched exactly, they only showed minimal deviation from each other. Our study also aligns with experimental research by Sydoff et al. [12] and Berthon et al. [6], who investigated the impact of cold walls on volume delineation. Berthon et al. used a phantom with similar sphere sizes and SBRs of up to 6.4 but did not directly evaluate VRT, although they found that thinner walls improve segmentation performance. Sydoff et al. developed wall-less [^18^F]-doped gelatin phantoms and demonstrated that the background dependence of the VRT was absent. Their study was limited to four SBRs (lowest: 5) and three sphere sizes (smallest: 15.6 mm). Tracer uniformity in the gelatin was not analyzed and leaching from the material was only assessed by inspection of PET data after overnight submersion.

At SBR values of 6 or more, our results show that the wall-related effects become negligible. However, lower SBR values are commonly observed in clinical PET imaging, e.g. in brain studies [27, 28]. For instance, the mean SBR of gliomas with regard to healthy tissue from the contralateral brain hemisphere was found to be approximately 2.5 for 320 patients that were scanned in-house after receiving [^18^F]FET PET [29]. The use of conventional phantoms for validation of segmentation methods could therefore lead to an overestimation of target volumes in clinically relevant cases. When only fillable phantoms are available, only high-contrast measurements should be performed to establish optimal segmentation thresholds.

3D printers that employ techniques such as SLA are well-suited for the production of calibration phantoms and offer the possibility of generating more complex geometries while producing rigid structures without requiring molds, unlike softer materials like gelatin and alginate. In the recent past, SLA printing has been successfully used as a method for manufacturing PET phantoms by introducing a variety of radioactive substances into the liquid resin [17–20]. We were able to confirm the feasibility of the printing workflow even for short-lived isotopes such as Fluorine-18. The resulting phantom accurately reflected the pre-defined activity distribution for our measurement and featured interchangeable parts with printed screw threads, enabling us to modify it for future experiments.

It is also crucial to acknowledge the limitations of radioactive 3D printing that we encountered during our study. For instance, the preparation of the phantom parts involves extensive manual handling which leads to an increased radiation exposure of the operator’s hands. This could be addressed by optimizing the preparation process and by using specialized tools that allow the operator to employ shielding, increase the distance to the printed object and to minimize the required handling time. In the case of our measurement, the ring dosimeter that the operator was wearing recorded an equivalent dose of only 1 mSv for the corresponding month, which is relatively low compared to the German legal limit of 500 mSv/year and regarding the fact that the operator conducted multiple measurements involving radiation in that month. The intrinsic difficulty of printing massive spheres presents another limitation that prolongs the handling time and reduces print quality. This could be resolved by using a Polyjet printer, which relies on a different printing technology and is considerably more expensive. Another solution to this could be to print two hemispheres that can be combined after the print. This would also shorten the printing time and eliminate the need for support structures. An additional potential limitation is the leaching of radioactivity from 3D printed parts. Läppchen et al. and Meier et al. introduced [^68^Ge] and [^99m^Tc] into the printing resin and found only minimal leaching, indicating near perfect source tightness [18, 19]. Gillett et al., who used [^18^F]FDG mixed with transparent Prusa resin, investigated this effect using a helix shape and found that 0.72% of the initial activity had leached into the surrounding water over a time span of 3 h [17]. The printing procedure that was followed in this work was mostly identical to their approach and the spheres were submerged for approx. 3.5 h. As spheres have a much lower surface-to-volume ratio than helical shapes, we therefore assume that leaching did not significantly affect our results. Furthermore, we analyzed the microscopic count distribution in the printed material and observed small regions with elevated counts. However, the resulting signal variation was reduced after applying the same Gaussian filter that was used for the PET reconstructions, yielding a standard deviation of only 2.4% relative to the mean. Our analysis of the PET voxel intensities from the inner 27 mm diameter sphere regions also showed that the coefficient of variation was generally very low and only differed by less than 0.5 percentage points between the sphere types. The Kolmogorov–Smirnov test did not indicate a statistically significant difference and the Kullback–Leibler divergence was near zero, suggesting virtually identical distributions. Elmoujarkach et al. [20], who followed a similar printing procedure, achieved a highly uniform distribution using [^18^F]FDG. Thus, we can expect that any inhomogeneity which might have formed in the resin did not affect homogeneity on the PET image voxel level and that the activity distribution in the resin was sufficiently uniform to achieve a constant SBR. Finally, it should be noted that 3D printed materials have physical properties that can differ from the properties of fillable phantoms. In our experiment, the SLA resin had a higher density and the CT images showed higher HU values for the wall-less spheres compared to water. It may therefore be useful to carry out further measurements to assess the attenuation properties of these materials or to use materials that have already been characterized [30].

Future research should aim to address the aforementioned limitations and explore the introduction of different radionuclides, such as Germanium-68 [19], into the printing material to manufacture longer-lived phantoms that can be used multiple times. Each type of resin and radiopharmaceutical should be examined individually to ensure uniform distribution and long-term stability. For radiopharmaceuticals that are provided as aqueous solutions, such as [^18^F]FDG, resins with increased hydrophilicity might be beneficial. Moreover, the capability of 3D printing to generate radioactive objects with complex geometries and irregular shapes should be exploited.

## Conclusions

We found that SLA 3D printed wall-less phantoms facilitate the validation of threshold-based segmentation methods in PET by eliminating the SBR dependance of the VRT, outperforming conventional fillable phantoms. Our study, together with the prior research, underlines the replicability and versatility of the 3D printing approach, offering a promising avenue for the development of phantoms that can be customized for a variety of research and clinical needs, such as the development of new PET systems, due to its ease of use and affordability. By providing a reliable and contrast-independent method for standardized assessment of segmentation methods, particularly in low-contrast scenarios common in clinical routine, this technology could contribute to improving the precision of target volume delineation for patient diagnostics and treatment planning. Moreover, the adoption of 3D printed phantoms can facilitate personalized medicine approaches. By allowing for the customization of phantoms to replicate patient-specific tumors or anatomical structures, 3D printing technology can contribute to the development of tailored imaging and treatment strategies that address the unique characteristics of individual patients.

## Supplementary Information

Supplementary file1.

## Data Availability

All data generated or analyzed during this study are included in this published article and its supplementary information files. The datasets used and/or analyzed during the current study are available from the corresponding author on reasonable request.

## Abbreviations

CAD: Computer-aided design
CT: Computed tomography
CV: Coefficient of variation
FDG: Fluorodeoxyglucose
HU: Hounsfield units
NEMA: National electrical manufacturers association
OSEM: Ordered subset expectation maximization
PET: Positron emission tomography
pp: Percentage points
SBR: Signal-to-background ratio
SLA: Stereolithography
VOI: Volume of interest
VRT: Volume-reproducing threshold
