# Supplementary material for: 3D printing of radioactive wall-less PET phantoms improves threshold-based target delineation and quantification
Source: EJNMMI Phys. 2025 Jun 6;12:53. doi: 10.1186/s40658-025-00768-x (PMC12144006; doi:10.1186/s40658-025-00768-x)
Supplement: Supplementary file 1 — Supplementary file1. [file 40658_2025_768_MOESM1_ESM.pptx]

## Slide 1
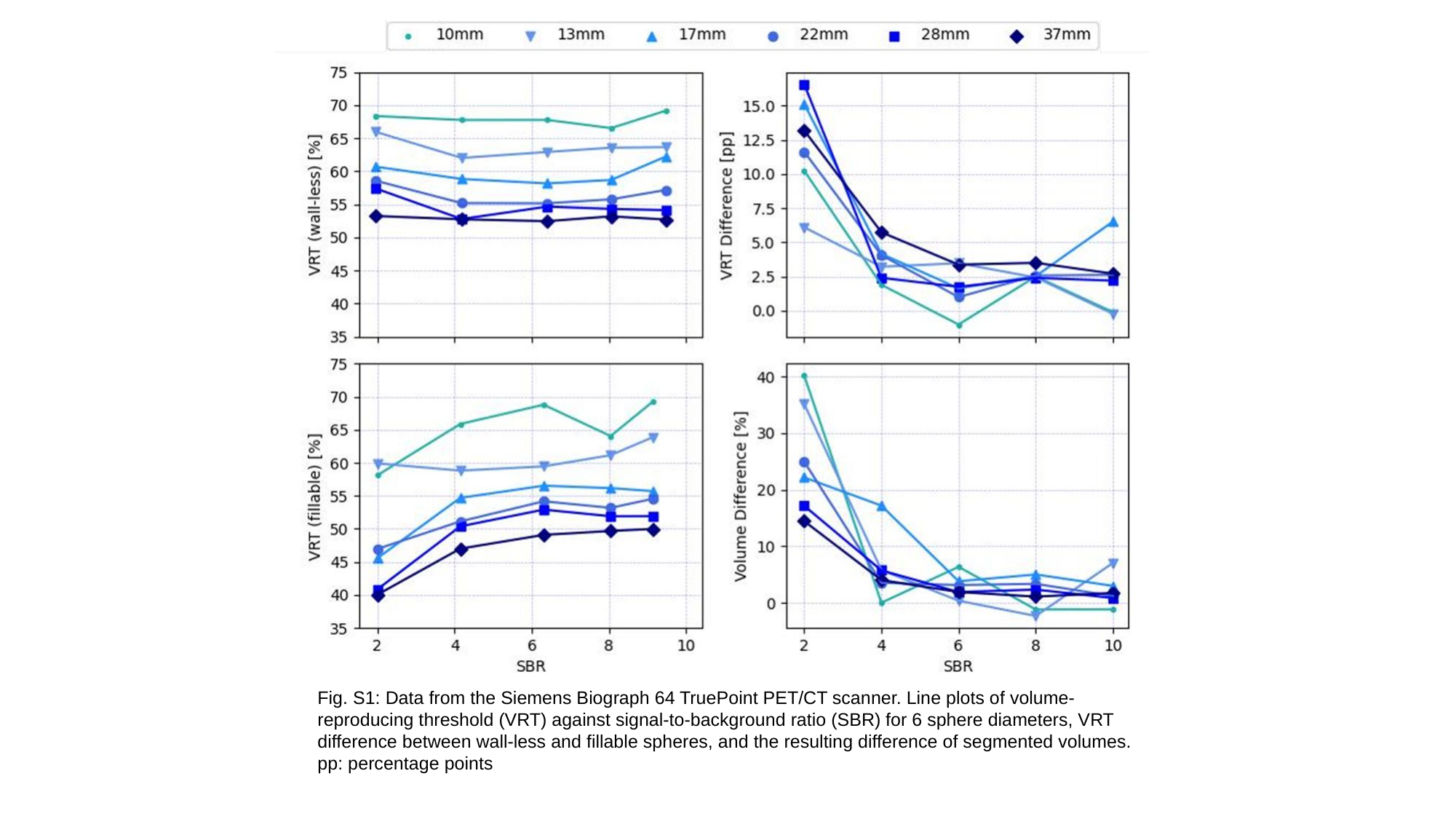

Fig. S1: Data from the Siemens Biograph 64 TruePoint PET/CT scanner. Line plots of volume-reproducing threshold (VRT) against signal-to-background ratio (SBR) for 6 sphere diameters, VRT difference between wall-less and fillable spheres, and the resulting difference of segmented volumes. pp: percentage points
